# EIF4A inhibition targets bioenergetic homeostasis in AML MOLM-14 cells in vitro and in vivo and synergizes with cytarabine and venetoclax

**DOI:** 10.1186/s13046-022-02542-8

**Published:** 2022-12-09

**Authors:** Katie Fooks, Gabriela Galicia-Vazquez, Victor Gife, Alejandro Schcolnik-Cabrera, Zaynab Nouhi, William W. L. Poon, Vincent Luo, Ryan N. Rys, Raquel Aloyz, Alexandre Orthwein, Nathalie A. Johnson, Laura Hulea, Francois E. Mercier

**Affiliations:** 1 Lady Davis Institute for Medical Research, Montreal, Canada; 2 Department of Medicine, McGill University, Montreal, Canada; 3 Maisonneuve-Rosemont Hospital Research Centre, Montreal, Canada; 4 Present Address: Département de Biochimie et Médecine Moléculaire, Université de Montréal, Montreal, Canada; 5 Department of Physiology, McGill University, Montreal, Canada; 6 Gerald Bronfman Department of Oncology, McGill University, Montreal, Canada; 7 Present Address: Department of Radiation Oncology, Emory School of Medicine, Atlanta, USA; 8 Département de Médecine, Université de Montréal, Montreal, Canada

**Keywords:** AML, eIF4A, BCL2, MCL1, BCL-XL, mTORC1, Metabolism, ROS, Bioenergetics, araC, Venetoclax

## Abstract

**Background:**

Acute myeloid leukemia (AML) is an aggressive hematological cancer resulting from uncontrolled proliferation of differentiation-blocked myeloid cells. Seventy percent of AML patients are currently not cured with available treatments, highlighting the need of novel therapeutic strategies. A promising target in AML is the mammalian target of rapamycin complex 1 (mTORC1). Clinical inhibition of mTORC1 is limited by its reactivation through compensatory and regulatory feedback loops. Here, we explored a strategy to curtail these drawbacks through inhibition of an important effector of the mTORC1signaling pathway, the eukaryotic initiation factor 4A (eIF4A).

**Methods:**

We tested the anti-leukemic effect of a potent and specific eIF4A inhibitor (eIF4Ai), CR-1-31-B, in combination with cytosine arabinoside (araC) or the BCL2 inhibitor venetoclax. We utilized the MOLM-14 human AML cell line to model chemoresistant disease both in vitro and in vivo. In eIF4Ai-treated cells, we assessed for changes in survival, apoptotic priming, de novo protein synthesis, targeted intracellular metabolite content, bioenergetic profile, mitochondrial reactive oxygen species (mtROS) and mitochondrial membrane potential (MMP).

**Results:**

eIF4Ai exhibits anti-leukemia activity in vivo while sparing non-malignant myeloid cells. In vitro, eIF4Ai synergizes with two therapeutic agents in AML, araC and venetoclax. EIF4Ai reduces mitochondrial membrane potential (MMP) and the rate of ATP synthesis from mitochondrial respiration and glycolysis. Furthermore, eIF4i enhanced apoptotic priming while reducing the expression levels of the antiapoptotic factors BCL2, BCL-XL and MCL1. Concomitantly, eIF4Ai decreases intracellular levels of specific metabolic intermediates of the tricarboxylic acid cycle (TCA cycle) and glucose metabolism, while enhancing mtROS. In vitro redox stress contributes to eIF4Ai cytotoxicity, as treatment with a ROS scavenger partially rescued the viability of eIF4A inhibition.

**Conclusions:**

We discovered that chemoresistant MOLM-14 cells rely on eIF4A-dependent cap translation for survival in vitro and in vivo. EIF4A drives an intrinsic metabolic program sustaining bioenergetic and redox homeostasis and regulates the expression of anti-apoptotic proteins. Overall, our work suggests that eIF4A-dependent cap translation contributes to adaptive processes involved in resistance to relevant therapeutic agents in AML.

**Supplementary Information:**

The online version contains supplementary material available at 10.1186/s13046-022-02542-8.

## Background

Acute myeloid leukemia (AML) is an aggressive hematological cancer that results from uncontrolled proliferation of myeloid cells blocked in terminal differentiation [1]. The standard of care for non-promyelocytic subtype AML patients without significant medical comorbidities involves myeloablative chemotherapy with cytarabine (araC) and daunorubicin, often followed by hematopoietic stem cell transplantation. Improved understanding of the genetic and epigenetic landscape of AML has enabled the development of targeted therapies for recurrent oncogenic mutations, such as those affecting the cytokine transmembrane receptor Fms-like tyrosine kinase 3 (FLT3), present in 30% of cases, and isocitrate dehydrogenase (IDH1/2), present in 20% of cases [2–5]. These inhibitors can improve clinical responses to chemotherapy or can be utilized as palliative monotherapy [6, 7]. Alternatively, venetoclax targets the antiapoptotic protein B-cell lymphoma 2 (BCL2), which is often overexpressed in AML [8, 9]. Combinations of venetoclax plus hypomethylating agents or low-dose of araC are used to treat patients unfit for chemotherapy [4, 10]. Noteworthy, there is no cure for most AML patients, as resistance and relapse often arise, thereby leading to a dismal outcome for over 70% of patients within 5 years after diagnosis [11].

**Fig. 1:**
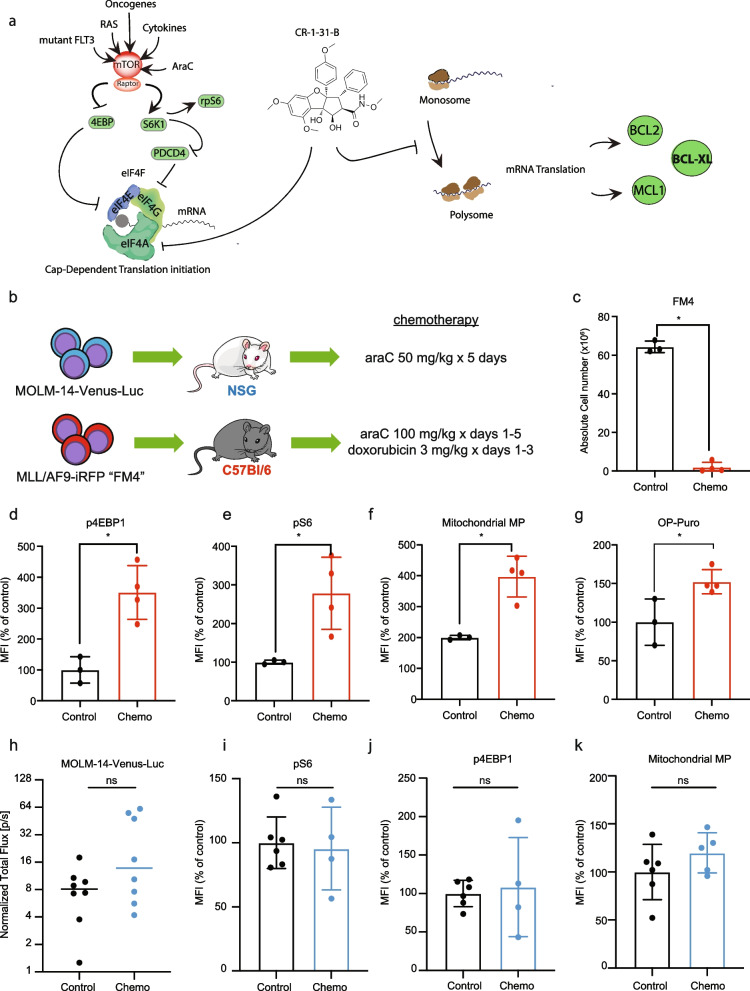
Chemoresistant AML cells show increased mTORC1 activity and mitochondrial function. **a** In AML, hyperactive mTORC1 signaling is driven by oncogenic signaling (i.e. mutated RAS, FLT3), chemotherapy and microenviromental cues such as cytokines [18–26]. mTORC1 is a multimeric complex containing the catalytic subunit mTOR serine/threonine kinase, and scaffolding unit Raptor. Two key mTORC1 substrates that regulate cap-dependent translation initiation are the eukaryotic initiation factor (eIF) 4E-binding proteins (4E-BPs) and the ribosomal protein S6 kinase 1 (S6K1). mTORC1 phosphorylation inhibits 4E-BPs, which are negative regulators of eIF4E. Active S6K1 phosphorylates rpS6 and the programmed cell death protein 4 (PCD4), a negative regulator of eIF4A. eIF4E cap-binding protein, eIF4G scaffolding protein and eIF4A RNA helicase are subunits of eIF4F, the rate-limiting complex in cap-dependent translation initiation. Activation of mTORC1 releases eIF4E and eIF4A from their negative regulators, 4EBPs and PDCD4, respectively, allowing the assembly of eIF4F. Proposed mechanisms for CR-1-31-B action include (i) direct inhibition of translation of the target mRNA by increasing eIF4A1:RNA binding (clamping) and (ii) depletion of the limited eIF4F pool available for ribosome recruitment leading to a trans-inhibitory effect toward mRNAs that are not directly affected by clamping [32, 33]. Among eIF4A targets are the BCL2 family members BCL-2, MCL-1 and BCL-XL [34]. **b** NSG or C57BL/6 J mice were transplanted with MOLM-14-Luc cells or FM4 cells, respectively. NSG mice were treated with 50 mg/kg araC injected intraperitoneally (i.p.) daily for 5 days. C57BL/6 J mice were treated with 100 mg/kg araC i.p. daily for 5 days with 3 mg/kg doxorubicin i.p. daily concomitantly from days 1-3. Analyses shown in **c**-**k** were performed at day 8 post treatment initiation. **c** Mean and SEM of total FM4 cells (y-axis) in bone marrow of C57BL/6 J mice treated with vehicle (Control, *n* = 3) or chemotherapy (*n* = 4). Significant differences were assessed by two-tailed unpaired t-test, *p < 0.05. **d**-**g** Chemoresistant FM4 AML cells were analyzed by flow cytometry to assess for differences on **d** p4E-BP, **e** pS6, **f** mitochondrial membrane potential (Mitochondrial MP) using TMRE and **g** OP-Puro incorporation. The y-axis represents the median fluorescence intensity and the bars, the mean values and SEM. Significant differences were assessed by two tailed unpaired t-test, *p < 0.05. **h** AML growth in NSG mice was assessed by in vivo bioluminescence imaging at days 1 and 8. Tumor burden on day 8 was normalized to tumor burden on day 1 (y-axis). The bar represents the mean value, ns indicates *p* > 0.05. **i**-**k** Chemoresistant MOLM-14 were analyzed by flow cytometry to assess for differences on **i** p4E-BP, **j** pS6, **k** Mitochondrial MP using TMRE. The y-axis represents the median fluorescence intensity and the bars, the mean values and SEM. Significant differences were assessed by two-tailed unpaired t-test; ns *p* > 0.05; MFI: mean fluorescence intensity. Data related to Fig. 1 are shown in Supp. Fig. 1

Mammalian target of rapamycin complex 1 (mTORC1) signaling integrates nutritional, environmental, and intracellular cues [12–14] to coordinate mitochondrial activity, metabolic fluxes and proliferation [15–17]. Noteworthy, mTORC1 signaling is often dysregulated in cancer. For instance, hyperactivation of mTORC1 in AML is driven by mutated oncogenic upstream drivers (e.g., *RAS*, *FLT3*) and by the in vivo AML microenvironment, contributing to disease progression and relapse [18–26]. Clinical strategies to target the hyperactivation of mTORC1 signaling have failed due to reactivation of the pathway through compensatory feedback loops [27]. An emerging strategy to curtail this setback involves targeting key mTORC1-driven targets and processes. Key to its role as master translational and metabolic regulator, mTORC1 coordinates the assembly of the eukaryotic initiation factor 4F (eIF4F), the rate-limiting heterotrimeric complex required for cap-dependent mRNA translation (Fig. 1a). Among eIF4F subunits, the mRNA helicase eIF4A was shown to drive the expression of key players in the metabolic adaptation of cancer cells including metabolite transporters, glycolytic enzymes and regulators of mitochondrial functions such as BCL2 family members [23, 28–31].

Metabolic adaptations in AML have been reported to entail rewiring of carbon and nitrogen fluxes that promote mitochondrial respiration and redox homeostasis [15, 35–38]. Studies using patient-derived xenograft (PDX) models revealed that araC-resistant AML cells overexpress genes driving OX/PHOS [39, 40], whereas murine syngeneic AML models have shown that residual cells increase the uptake of glutamine and aspartate to fuel OX/PHOS and synthesize pyrimidine and glutathione [41], which may represent adaptations required to survive in that context. The role of the mTORC1/eIF4F axis as an orchestrator of metabolic rewiring in AML relapse is not known. Since preliminary studies have highlighted the suitability of eIF4A as a therapeutic target in different hematological malignancies both in vitro and in vivo [42, 43], we sought to evaluate the effect of the eIF4A inhibitor (eIF4Ai) CR-1-31-B on AML survival, mRNA translation and mitochondrial function, both as single agent and in combination with araC [44]. CR-1-31-B is a silvestrol-related rocaglate compound with promising pharmacokinetic properties in mice [45]. Although EIF4A1 is essential for normal cell viability, prior work using genetic and pharmacological models indicate that a therapeutic window between normal and malignant cells exist for partial inhibition of the translation initiation machinery [31, 46].

In this study, we leveraged experimental models of AML, murine and human, that are characterized by mTORC1 dysregulation to investigate the contribution of eIF4A as a downstream effector. The murine syngeneic model of AML driven by the human KMT2A/MLLT3 translocation [47] depends on transient mTORC1 activity for disease initiation or relapse after chemotherapy, as shown using in vivo CRISPR screening [48] or fluorescent reporters [18]. Using this model, we found that mTORC1 activation in the context of relapse is associated with increased mRNA translation and mitochondrial activity in vivo. Next, we validated the therapeutic effect of eIF4Ai in the chemoresistant MOLM-14 human AML cell line, which harbors a KMT2A/MLLT3 translocation and a FLT3-ITD mutation that constitutively activates mTORC1 signaling in vitro [12]. The MOLM-14 AML cell line is ideally suited for this study as it was derived from a patient who did not respond to chemotherapy [49] and has also been described as chemoresistant in xenograft models in association with enhanced OX/PHOS and metabolic rewiring [39]. Our results indicate that pharmacological eIF4Ai using CR-1-31-B in MOLM-14 cells impairs mitochondrial and bioenergetic functions, synergizes with venetoclax and araC in vitro, and reduces the expression of the anti-apoptotic proteins BCL2, BCL-XL and MCL1. Importantly, we confirmed that CR-1-31-B impairs MOLM-14 cell growth in vivo and is overall well tolerated by mice, with minimal systemic toxicity except for a partial decrease in B-lymphopoiesis.

## Methods

### Cells lines

The AML cell lines U937 and KG1a were ordered from the American Type Culture Collection (ATCC) (U937 and KG1a). The human AML cell line MOLM-14 was a generous gift from the laboratory of Dr. David B. Sykes. The generation of the FM4 cell lines has previously been described [18]. MOLM-14, KG1a, and U937 cells were maintained in RPMI-1640 supplemented with 10% FBS (fetal bovine serum, Wisent Bio Products and Hyclone) and 100 units/ml penicillin/streptomycin at 37 °C with 5% CO_2_, and routinely tested for mycoplasma. The MOLM-14-Venus-Luciferase cell line was generated by lentiviral transduction with the pLenti CMV Puro LUC (w168-1) (a gift from Eric Campeau & Paul Kaufman; Addgene plasmid #17477) and the LeGO-V2 plasmid (a gift by Dr. Boris Fehse; Addgene plasmid #27340). Lentiviral particles were generated as previously described [50]. Briefly, 5 × 10^5^ MOLM-14 cells in 1 mL of culture media were then seeded into 6-well plates with 0.5 mL of pLenti CMV puro LUC lentivirus, 0.5 mL of LeGO-V2 lentivirus, and 4 μg/ml of polybrene. MOLM-14 cells were then infected at 1000 g for 1 h at 32 °*C. Venus*-positive cells were sorted using a FACSAria Fusion instrument, then selected with 10 μg/mL Puromycin (BioShop Canada). Stable luciferase expression was confirmed using the Promega GloMax 20/20 Luminometer and D-Luciferin (Cedarlane) prior to transplant. The identity of the MOLM-14-Venus-Luciferase cell line was confirmed using the ATCC cell line authentication service. The iRFP+ FM4 cell line was a generous gift from David Scadden and was maintained in RPMI-1640 supplemented with 10% FBS, penicillin/streptomycin, 6 ng/ml rmIL-3, and approximately 100 ng/ml SCF generated from a Chinese hamster ovary (CHO) cell line, as previously described [50].

### In vitro drug treatments

AraC and CR-1-31-B were obtained from MedChemExpress and resuspended in DMSO. Venetoclax was obtained from Selleckchem and resuspended in DMSO. Aliquots of stock solution of the drugs were stored at − 80 °C and diluted into working solutions immediately before use. MOLM-14 cells were plated at 0.5× 10^6^ cells/ml and treated with drug at the indicated concentrations or an equal volume of vehicle.

### Synergy analysis

MOLM-14 cells (5000-20000) were seeded in triplicates in 96-well plates and treated with increasing drug concentrations in pairs as indicated: araC (0-250 nM) or venetoclax (0-6.26 μM) with CR-1-31-1B (0-10 nM). Viability was assessed 24 h later using the CellTiter-Glo (Promega) luminescent viability assay. Readouts were normalized to the values in vehicle treated cells and analyzed with the SynergyFinder application using the Bliss model. The Bliss independence model assumes a stochastic process in which two drugs elicit their effects independently, and the expected combination effect can be calculated based on the probability of independent events [51]. Bliss score less than − 10: the interaction between two drugs is likely to be antagonistic; from − 10 to 10: the interaction between two drugs is likely to be additive; larger than 10: the interaction between two drugs is likely to be synergistic [52].

### *O*-propargyl-puromycin incorporation assay

We used the Click-iT Plus OP-Puro Kit (ThermoFisher Scientific) according to the manufacturer’s instructions. Briefly, 5 million MOLM-14 cells were treated for 48 h. Immediately after, cells were transferred to pre-warmed growth media containing 20 μM of O-propargyl-puromycin reagent and incubated for 30 min. Click-iT chemistry reactions were then performed following the manufacturer’s instructions and the samples analyzed on a BD LSR Fortessa cytometer.

### Polysome profiles

Polysome profiles were obtained as described before [31, 53, 54]. Briefly, MOLM-14 cells were treated with araC for 48 h and CR-1-31-B for 1 h. For the last 5 min, cycloheximide was added to the treatment media (final concentration 100μg/ml). Cells were lysed in hypotonic lysis buffer (5 mM Tris HCl pH 7.5, 2.5 mM MgCl2, 1.5 mM KCl, 100 μg/mL cycloheximide, 2 mM DTT, 0.5% Triton,0.5% Sodium Deoxycholate) and the cytoplasmic extracts were resolved on 5–50% sucrose gradients by centrifugation in an SW40 rotor at 150,000×g for 2 h. The absorbance at 260 nm was measured using a Triax™ Flow Cell instrument (BioComp, Canada). The results were plotted in R as previously [17] and the polysome/monosome (P/M) ratios calculated. Polysome to 80S ratios were calculated by comparing the area under the 80S peak and the combined area under the polysome peaks.

### Mitochondrial assays

Mitochondrial membrane potential and mtROS production were assessed by flow cytometry using Mitostatus TMRE (BD Biosciences) and MitoSOX (ThermoFisher) according to the manufacturer’s instructions. Five million MOLM-14 cells were treated for 48 h. Treated cells were incubated for 20 min with a final concentration of 50 nM TMRE and 2.5uM mitoSOX red following the manufacturer’s instructions. DAPI was added as a viability marker. Cells were analyzed by flow cytometry using a BD LSR Fortessa cytometer and analyzed using the FlowJo software.

### Extracellular flux

Oxygen consumption rate (OCR) and extracellular acidification rate (ECAR) were measured using Seahorse XFe96 instrument (Agilent Technologies, CA, USA) according to the manufacturer’s protocol [55]. MOLM-14 cells (750,000 cells/well) were seeded in 6-well plates. Six wells were used for each condition. Cells were treated for 48 h (araC) and/or 24 h (CR-1-31-B) and 150,000 cells/well were re-plated in 96-well Seahorse plates coated with Cell-Tak, as per the manufacturer’s instructions and bioenergetic profiles were obtained as described, using the following inhibitors (1 μM Oligomycin, 1.5 μM FCCP, 0.5 μM Rotenone/Antimycin A, 10 mM glucose, 50 μM 2-DG) [55]. Measurements for OCR and ECAR were conducted in an XFe96 Seahorse instrument and values were normalized to cell counts. Cellular mitochondrial and glycolytic ATP production were quantified as described [56]. Three independent experiments were performed.

### Dynamic BH3 profiling

BH3 profiling was carried out as previously described, using protocols adapted from Ryan et al. [57, 58]. Briefly, 10 million MOLM-14 cells in 20 mL of culture media were treated for 24 h. with 2.5 nM CR-1-31-B or vehicle control. After treatment, cells were stained using aqua live/dead (Invitrogen, #L34966). Following exposure to digitonin, cells were incubated with varying concentrations of BH3 peptides and inhibitors involved in apoptotic activation for 90 min. Moreover, cells were incubated with inhibitors targeting BCL2 (Venetoclax, AbbVie), MCL1 (S63845, SelleckChem) and BCL-XL (A-1331852, SelleckChem) or multiple BCL2 proteins (ABT-737, SelleckChem). After incubation, cells were fixed and stained overnight at 4 °C for cytochrome C (BD Biosciences, #558709). Retention of cytochrome C was measured using a Fortessa Flow Cytometer (BD Biosciences) where DMSO is a control for cytochrome C retention and alamethicin (ALA) is a control for cytochrome C release. BH3 results (cytochrome C release) are expressed as a percentage of the inverse of staining cytochrome C retention. Acquired data was normalized to DMSO in each experiment. Results from untreated conditions were subtracted from treated ones to calculate a change in response (“apoptotic priming”) upon 24 h exposure to CR-1-31-B. Four independent experiments were performed.

### GC/MS and stable isotope tracer analyses of cellular samples

MOLM-14 cells were treated with vehicle, araC or CR-1-31-B as indicated. Cells were then rinsed three times with 4 °C saline solution (9 g/L NaCl) and quenched with 600 μl 80% MEOH (− 0 °C). Membranes disruption was carried by sonication at 4 °C (2x10 min, 30 sec on, 30 sec off, high setting, Diagenode Bioruptor). Extracts were cleared by centrifugation (15,000 rpm, 10 min, 4 °C) and supernatants were transferred into new tubes containing 1 μl 800 ng/μl myristic acid-D_27_ (Sigma). Next, they were dried in a cold trap (Labconco) overnight at − 4 °C. Pellets were solubilized in 30 μl pyridine containing methoxyamine-HCl (10 mg/mL, Sigma) by sonication and vortex, and were incubated at RT for 20 min (methoximation). Samples were centrifuged (15,000 rpm, 10 min, RT) and the supernatants were transferred into glass vials containing MTBSTFA (70 μl, Sigma) for derivatization at 70 °C for 1 h. One μL was injected per sample for GC–MS analysis. GC–MS instrumentation and software were all from Agilent. GC–MS methods and analyses are as previously described [17]. Data analyses were performed using the Chemstation and MassHunter software (Agilent, Santa Clara, USA) from two independent experiments, each consisting of three technical replicates.

### BCL2, BCL-XL, MCL1 and mTORC1 signaling

MOLM-14 cells (500,000 cells/ml) were seeded into T-75 flasks, treated with vehicle, araC or CR-1-31-B as indicated. Samples were then washed and collected in PBS and cell pellets were immediately frozen at − 80 °C before being lysed in lysis buffer [50 mM Tris/HCL pH 7.4, 150 mM NaCl, 0.1%SDS, 1%NP-40, 0.5% Sodium Deoxycholate1X complete protease inhibitors (Roche), 1x PhosSTOP (Roche)]. Protein concentrations in cell extracts were determined using Pierce BCA Protein Assay Kit (Thermo Fisher Scientific). Proteins were resolved by SDS-PAGE, and then transferred to nitrocellulose membranes (Cytiva Amersham). The following primary antibodies were used: anti-β-actin (1:5000 dilution, Sigma #A5441); anti-BCL-2 (D55G8, Cell Signaling #4223), anti-BCL-XL (54H6, Cell Signaling #2764S), anti-MCL1 (D35A5, Cell Signaling #5453) (all at 1:1000 dilution). Horseradish peroxidase-conjugated anti-rabbit IgG and anti-mouse IgG antibodies were from Cell Signaling. Signals were revealed by chemiluminescence (ECL, Perkin Elemer, from FroggaBio) and Thermo Fisher Scientific Azur c600 instrument.

Changes in p4E-BP and pS6 phosphorylation status were assessed in fixated cells recovered from the bone marrow (BM) of AML murine models. AML cells recovered from mice were washed and incubated with BD Fixable Viability Stain 700, fixed in 37% formaldehyde and permeabilized in ice-cold methanol. Samples were resuspended in PBS and incubated in anti-pS6 (1:250, Cell Signaling Technology; Alexa Fluor 594 conjugate), anti-p4E-BP1 (1:1000, Cell Signaling Technology; Alexa Fluor 488 conjugate) and analyzed by flow cytometry (BD LSR Fortessa Cytometer).

### MOLM-14 xenograft murine model

All in vivo experiments were approved by the animal care committee of McGill University. All mice were housed at the animal care facilities and had ad libitum water and food access and were maintained on a 12-h light day cycle, mean temperature 22.5 ± 1.5 °C, and 22–28% humidity. 8-12-week-old male NOD.Cg-Prkdc^scid^IL2Rg^tm1Wjil^/SzJ (NSG) mice were sub-lethally irradiated with 2.5 Gy 24 h prior to transplant and given enrofloxacin in their drinking water for 14 days. Venus- and luciferase-expressing MOLM-14 cells (MOLM-14-Luc) were washed twice in PBS, re-suspended in PBS at 1 × 10^7^ cells/ml, and 200 uL (2 × 10^6^) cells were intravenously injected. Mice were assessed for engraftment at 10-12 days post-transplant by bioluminescent imaging using a Perkin Elmer IVIS Spectrum (detailed below). Mice were treated with 0.20 mg/kg CR-1-31-B i.p. daily for 4 to 7 days; CR-1-31-B was diluted in 5.2% TWEEN 80/5.2% PEG400. As controls, mice were treated with 5.2% TWEEN 80/5.2% PEG400 vehicle alone. On day 5 or 8, mice were re-imaged.

### FM4 syngeneic murine model

C57BL/6 J founders were obtained from Charles River Laboratories and bred at the Lady Davis Institute. 8-12-week-old male C57BL/6 J mice were sub-lethally irradiated with 4.5 Gy 24 h before transplant. FM4 cells were washed twice in PBS, re-suspended in PBS at 2.5 × 10^7^ cells/ml, and 200 μL (5 × 10^6^) cells were intravenously injected. To quantify engraftment starting at 14 days post-transplant and weekly thereafter, 50-100 μl of peripheral blood was collected from the saphenous vein, the red blood cells were lysed using Gibco ACK Lysing Buffer, and iRFP+ FM4 cells were quantified as percentage of white blood cells (WBC) using a BD Fortessa flow cytometer. When FM4 cells reached a level greater than 1% of WBC, representative of at least 50% BM burden, C57BL/6 J mice were treated with 100 mg/kg AraC i.p. daily for 5 days with 3 mg/kg doxorubicin i.p. daily concomitantly from days 1-3. AraC and doxorubicin were diluted in sterile PBS. As controls, mice were treated with vehicle alone. On day 8, mice were euthanized and mononuclear cells were purified from BM using Ficoll-Paque density centrifugation.

### CR-1-31-B toxicity in vivo

Healthy C57BL/6 J mice were treated with 0.20 mg/kg CR-1-31-B i.p. daily for 7 days. On day 8, mice were euthanized and mononuclear cells were purified from BM using Ficoll-Paque as indicated above. Spleens were crushed and treated with ACK lysing buffer. CR-1-31-B was diluted in 5.2% TWEEN 80/5.2% PEG400.

### Analysis of hematopoietic subpopulation

The analysis of hematopoietic populations was performed in the BM of vehicle- and CR-1-31-B-treated healthy C57BL/6 J mice with 0.20 mg/kg CR-1-31-B i.p. daily for 7 days. Mice were euthanized at day 8 and mononuclear cells were purified from BM by Ficoll-Paque density centrifugation. Populations of interest were analyzed by flow cytometry utilizing specific antibodies as follows:

#### Lineage cocktail primary FC antibodies

Biotin anti-mouse/human CD45R/B220 Antibody clone RA3-6B2, anti-mouse CD8a Antibody clone 53-6.7, Biotin anti-mouse CD4 Antibody clone RM4-5, anti-mouse TER-119/Erythroid Cells Antibody clone TER-119, anti-mouse CD3 Antibody clone 17A2, Biotin anti-mouse Ly-6G/Ly-6C (Gr-1) and Biotin anti-mouse/human CD11b Antibody Clone M1/70 (BioLegend).

#### Lineage cocktail secondary antibodies

FC Streptavidin, Pacific Orange™ conjugate (Thermo Fisher).

#### HSPC cocktail FC antibodies

Brilliant Violet 785™ anti-mouse CD117 (c-kit) clone 2B, Brilliant Violet 785™ anti-mouse CD117 (c-kit) clone 2B8, Brilliant Violet 421™ anti-mouse Ly-6A/E (Sca-1) Antibody Clone D7 BV421 (BioLegend); CD135 (Flt3) Monoclonal Antibody (A2F10), PerCP-eFluor 710, PerCP-eFluor 710 (eBioscience); PE Rat anti-Mouse CD34 Clone RAM34 (RUO), Ms. CD16/CD32 BV605 2.4G2 and Ms. CD127 BUV737 SB/199 (BD Biosciences).

#### Lineage cocktail FC antibodies

APC/Cyanine7 anti-mouse Ly-6G/Ly-6C (Gr-1) clone RB6-8C5, Brilliant Violet 421™ anti-mouse/human CD11b clone M1/70, Brilliant Violet 785™ anti-mouse/human CD45R/B220 clone RA3-6B2, PE/Cy7 anti-mouse CD8a clone 53-6.7 and APC anti-mouse CD4 (BioLegend). The visualization of the flow cytometry data was performed with the “pseudocolor” or “contour” modes of Flowjo analysis software v10, as shown in Supp. Figs. 1, 3-5. Gating of the hematopoietic subsets was performed using internal negative control cell populations, as defined by the “Guidelines for the use of flow cytometry and cell sorting in immunological studies” [59, 60], and was applied uniformly to all samples analyzed.

### In vitro colony-forming assays

Mononuclear cells were purified from whole-mouse BM using Ficoll-Paque density centrifugation. For non-AML experiments, 2 × 10^4^ cells were plated into hematopoietic progenitor-supporting MethoCult M3434 methylcellulose medium and 1 × 10^5^ cells were plated into MethoCult M3630 B-progenitor cell supporting methylcellulose. Colonies were propagated in culture for 7 and 12 days respectively, before being counted.

### In vivo bioluminescent imaging

At 10-12 days post-transplant, NSG mice were given one i.p. injection of 150 mg/kg D-luciferin in PBS. Mice were then anesthetized with 2% isoflurane and imaged using the IVIS Spectrum in vivo system. Kinetic curves were optimized for the MOLM-14-Luc cell line prior to treatment by imaging transplanted mice every 2 min for 45 min beginning 1 min after D-luciferin injection. Images were captured using Living Image analysis software and total flux values (photons/second) were determined using regions of interest (ROI) of the same size for each mouse. The total flux values of each image over the 45-min time course were plotted and the point of maximum luminescence was chosen as the optimal time point for imaging. Mice were imaged on days 1 and 8 of treatment and the final total flux value for each mouse was calculated by normalizing to the day 1 value.

### Data analysis

All flow cytometry experiments were analysed using FlowJo v10 software. The experimental results were analyzed and represented using GraphPad Prism. All tests were performed with α and statistical power equal or greater than 0.5 and 90%, respectively.

## Results

### Chemoresistant AML cells show increased mTORC1 activity and mitochondrial function

Since AML chemoresistance is associated with mitochondrial function, and mTORC1 promotes mitochondrial biogenesis and function in other contexts [37], we sought to confirm the link between chemotherapy response, mTORC1 activation, and mitochondrial activity in vivo using two well-characterized models of AML: 1) the chemoresistant human AML cell line MOLM-14, transplanted into NSG mice, and 2) the murine C57Bl/6 syngeneic AML model “FM4”, which transiently responds to chemotherapy but rapidly relapses [18] (Fig. 1b). Both models are driven by the human MLL/AF9 (KMT2A/MLLT3) translocation and the MOLM-14 cell line carries, in addition, an internal duplication of the FLT3 receptor tyrosine kinase (FLT3-ITD). Each murine strain was treated with chemotherapy doses determined by tolerability: 50 mg/kg of araC daily for 5 days for the NSG strain [39, 40], and daily doses of 100 mg/kg of araC for 5 days and 3 mg/kg of doxorubicin for 3 days for C57Bl/6, given concomitantly for days 1-3 [61]. As previously shown [18], chemo-response of FM4 AML cells at the nadir of 8 days (Fig. 1c, Supp. Fig. 1a) is associated with hyperactivation of targets downstream of mTORC1, 4E-BP1 (Fig. 1d) and S6 (Fig. 1e, Supp. Fig. 1b). Consistent with the known functions of mTORC1, mitochondrial activity, as measured by TMRE dye that is sequestered in polarized mitochondria, was increased in chemotherapy-residual FM cells (Fig. 1f, Supp. Fig. 1b), and incorporation of O-propargyl-puromycin was increased (Fig. 1g), indicative of elevated rates of protein synthesis. Overall, these results suggest that mTORC1 hyperactivation in chemotherapy-residual cells, known to drive relapse in this murine AML model, leads to increased synthesis of proteins and mitochondrial activity. In contrast, as shown by others [39], MOLM-14 AML cells did not significantly respond to chemotherapy in vivo at day 8 (Fig. 1h). Treatment with araC failed to induce an mTORC1 response at the level of S6 and 4E-BP1 (Fig. 1i, j) and there were no changes in mitochondrial polarization (Fig. 1k). Causes for the different chemo-responsiveness of the FM4 and MOLM14 models include different sensitivities to araC (in vitro IC_50_ values of 0.955 μM for MOLM-14 and 0.163 μM for FM4, data not shown) or the lower dose of araC administered to NSG mice, limited by tolerability. The absence of an mTORC1 response in MOLM14 cells could be related to insensitivity to araC or constitutive activation of the pathway downstream of the FLT3-ITD mutation [12, 49]. The chemoresistance of MOLM14 cells has previously been linked to their intrinsically elevated rate of oxidative phosphorylation [39]. This led us to investigate the links between mTORC1-driven protein synthesis, oxidative phosphorylation, and AML cell survival, and whether inhibition of mTORC1-driven protein synthesis could play a therapeutic role in this chemoresistant model.

### EIF4A inhibition impairs bioenergetic fitness and survival in a ROS-dependent manner

**Fig. 2:**
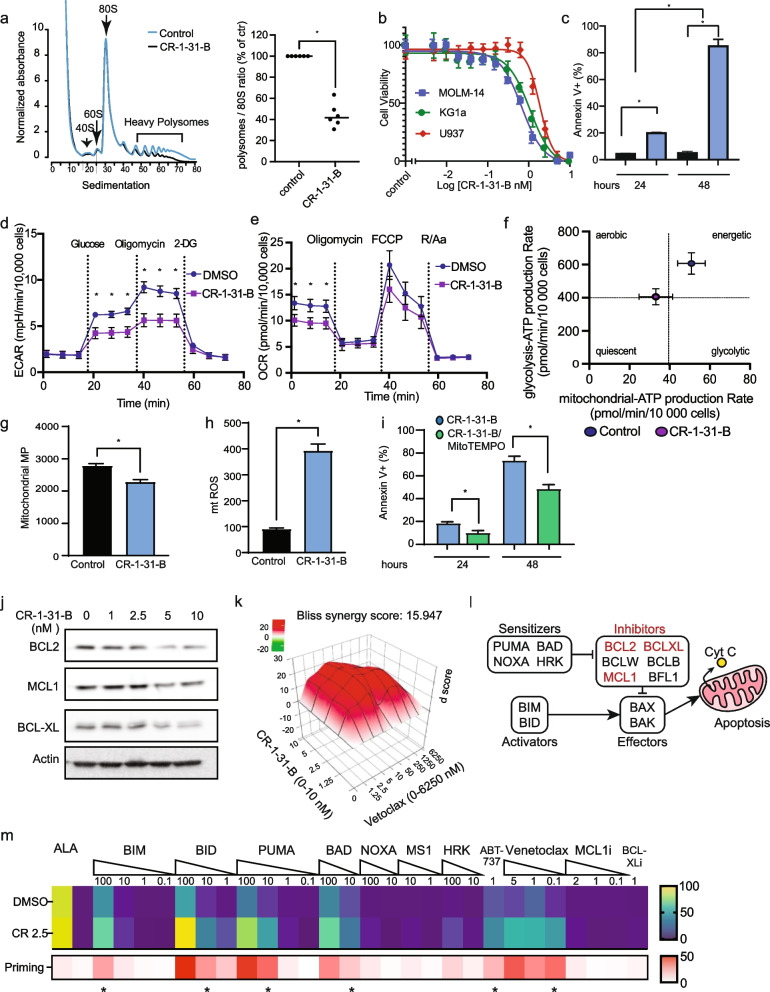
EIF4A inhibition impairs bioenergetic fitness and survival in a ROS-dependent manner. CR-1-31-B halts mRNA translation in MOLM-14 cells: **a** (left) MOLM-14 cells were treated for 1 h with vehicle or 10 nM CR-1-31-B. Sub-polysome, light-, and heavy-polysome fractions were obtained by ultracentrifugation using 5–50% sucrose gradients. Positions of 40S and 60S ribosomal subunits, monosomes (80S), and polysomes in the absorbance profiles (y-axis, 254 nm) are shown. The ratio between polysomal/monosomal fractions is shown in the right panel. CR-1-31-B is cytotoxic to AML cells: **b** KG1A, U937 and MOLM-14 cells were treated with CR-1-31-B (0-10 nM) and viability was determined after 48 h using the CellTiter-Glo assay. **c** The percentage of apoptotic cells (Annexin V^+^) after treatment with vehicle or 2.5 nM CR-1-31-B for 24 and 48 h was determined using flow cytometry. CR-1-31-B reduces bioenergetic capacity: MOLM-14 cells were treated with vehicle or 2.5 nM CR-1-31-B for 24 h. **d** Oxygen consumption rate (OCR) and **e** Extracellular acidification rate (ECAR) profiles were obtained using the Seahorse XFe technology. **f** OCR and ECAR data were used to estimate the rate of ATP synthesis by mitochondrial respiration or glycolysis and the bioenergetic profiles. Data were normalized by cell counting (10,000 cells/condition). CR-1-31-1B-dependent mitochondrial ROS is cytotoxic in MOLM-14 cells: MOLM-14 cells were treated with vehicle or 2.5 nM CR-1-31B for 48 h. **g** Mitochondrial membrane potential (mitochondrial MP) and **h** Mitochondrial superoxide content (mtROS) were quantified by flow cytometry using TMRE and mitoSOX respectively in viable cells. a.u. arbitrary units. **i** MOLM-14 cells were treated for 24 and 48 h with 2.5 nM CR-1-31B alone or after pre-incubation with 10 μM mitoTEMPO. The percentage of apoptotic (Annexin V^+^) was assessed by flow cytometry normalized to vehicle-treated cells. EIF4A inhibition reduces BCL2 and MCL1 expression levels and synergizes with venetoclax: **j** MOLM-14 cells were treated as indicated for 24 h. BCL2, BCL-XL and MCL1 levels were assessed by western blot analysis. β-actin was used as loading control. **k** MOLM-14 cells were treated with CR-1-31-B (0-10 nM) in combination with venetoclax (0-6.25 μM) for 24 h. Viability data was obtained using CellTiter-Glo and synergy between the drugs was assessed using the SynergyFinder application. Data related to Fig. 2j are shown in Supp. Fig. 2a. **l** Members of the BCL2 family of proteins consist of pro-apoptotic activators and sensitizers, and anti-apoptotic inhibitors, which modulate the activity of effectors through BH3-domain interactions. Disruption of the balance of pro-apoptotic and antiapoptotic BCL2 family proteins results in BAX/BAK activation, cytochrome C release and apoptosis [57, 58]. Highlighted in red are apoptotic inhibitors affected by eIF4A in MOLM-14 cells. **m** The effect of CR-1-31-B on cytochrome C release was measured as described using flow cytometry [58]. The upper heatmap represents the percentage of cell undergoing cytochrome C release after treatment with DMSO or 2.5 nM CR-1-31-B alone or in combination with the apoptotic activators, sensitizers or inhibitors as indicated. The concentrations of the compounds are indicated in μM. Alamethecin (ALA) is a positive control that induces cytochrome C release in all cells independent of BAX or BAK. The lower heatmap represents apoptotic priming by 2.5 nM CR-1-31-B as calculated by the difference in the percentages of cells undergoing cytochrome C release between CR-1-31B and DMSO treated cells (0-50% scale). The asterisks indicate that CR-1-31-B enhances the percentage of cytochrome C releasing cells (*p* < 0.05) with respect to DMSO control, as assessed by t-test. Footnote. The bars or circles represent the mean values of at least 3 independent experiments plus/minus the standard error of the mean (SEM) for b, c, g-i or standard deviation for d-f. Comparisons between groups were assessed by One-way ANOVA followed by paired t-test with *p < 0.05. All functional analyses were performed by flow cytometry in viable DAPI-negative cells. Representative gating strategies for cytochrome C release and BH3 peptide/inhibitor targets are shown in Supp. Fig. 5

mTORC1-driven protein synthesis is dependent upon the activity of the eIF4F complex, in which eIF4A plays an essential function. To test the effect of protein synthesis inhibition, the rocaglate CR-1-31-B, a potent and specific eIF4Ai, was used. First, we validated the effect of CR-1-31-B on mRNA translation initiation by polysome profiling analysis. As previously described, 10 nM of CR-1-31-B for 1 h increased the monosome fraction (80S) while reducing the polysomal fraction [44]. The reduction of translation initiation by CR-1-31-B was estimated by the polysome/monosome ratio between vehicle and CR-1-31-B treated MOLM-14 cells [44] (Fig. 2a). These results indicate that CR-1-31-B reduces de novo protein synthesis at a low nanomolar concentration. Next, we assessed the effect of CR-1-31-B on the viability of 3 human AML cell lines, including MOLM-14 cells. Our results show that CR-1-31-B reduces viability by half (IC_50_) in all three cell lines in the nanomolar range (Fig. 2b). In addition, Annexin V staining revealed that CR-1-31-B strongly induces apoptosis in MOLM-14 cells in a time dependent manner (Fig. 2c).

To gain insight on into the role of eIF4A on metabolic rewiring in AML cells, we specifically investigated the effect of CR-1-31-B on two eIF4A-dependent processes, mitochondrial respiration and glycolysis [39, 62–64]. Using the Seahorse extracellular flux analyzer, we observed that CR-1-31-B affects both glycolysis and mitochondrial respiration of AML cells. Specifically, CR-1-31-B-treated MOLM-14 cells displayed significantly reduced glycolysis, glycolytic capacity and glycolytic reserve, suggesting that basal glycolytic activity is diminished as well as the ability of MOLM-14 cells to respond to increased glycolytic demand in the context of compromised oxidative phosphorylation (Fig. 2d). CR-1-31-B reduced as well basal mitochondrial respiration in MOLM-14 cells (Fig. 2e). Altogether, these results indicate that eIF4A supports bioenergetic metabolism in AML cells. In line with these observations, the rate of ATP synthesis was reduced by CR-1-31-B in MOLM-14 cells, indicating a shift to a lower bioenergetic state (Fig. 2f). Consistent with bioenergetic stress, CR-1-31-B reduced significantly mitochondrial membrane potential (MMP; Fig. 2g). In line with a previous observation in pancreatic cancer cells [31], CR-1-31-B significantly enhanced mtROS levels in MOLM-14 cells (Fig. 2h). Notably, a ROS scavenger (MitoTEMPO) reduced CR-1-31-B-induced cell death by a half in MOLM-14 cells (Fig. 2i), therefore suggesting that oxidative stress contributes to CR-1-31-B cytotoxicity. Because the main source of mtROS is linked to mitochondrial respiration, which is reduced upon CR-1-31-B treatment, our results suggest that elevated mtROS in CR-1-31-B-treated MOLM-14 cells is probably associated with reduced redox buffering capacity.

### EIF4A inhibition reduces BCL2 expression and synergizes with venetoclax

Next, we validated the role of eIF4A in facilitating the expression of BCL2, BCL-XL and MCL1 in MOLM-14 cells, since these antiapoptotic proteins sustain mitochondrial fitness under stress and mediate therapeutic resistance in AML [10, 65]. Treatment of MOLM-14 cells with CR-1-31-B reduced significantly BCL2, BCL-XL and MCL1 in a dose-dependent manner (Fig. 2j, Supp. Fig. 2a). Furthermore, treatment of MOLM-14 cells with both CR-1-31-B and the BCL2 inhibitor venetoclax reduced viability of MOLM-14 cells in a synergistic manner, with a Bliss delta score of 16 (Fig. 2k). It is possible that CR-1-31-B-mediated reduction of BCL2 and family members MCL1 and BCL-XL contributes to the synergistic effect, since MCL1 was shown to contribute to venetoclax resistance, both in preclinical models in vivo and in AML patients [10, 66].

### EIF4A inhibition increases apoptotic priming of MOLM-14 cells

To gain insight into the mechanisms involved in the modulation of apoptosis by eIF4Ai, we performed dynamic BH3 profiling of MOLM-14 cells treated with 2.5 nM CR-1-31-B or vehicle. Dynamic BH3 profiling can identify the key apoptotic blocks of cancerous cells, by incubating with pro-apoptotic BH3 peptides or inhibitors of anti-apoptotic proteins, and then quantifying release of cytochrome C from the mitochondria (model in Fig. 2l). Our results indicate that MOLM-14 cells are competent to undergo apoptosis (e.g. functional BAX/BAK pore complex) since the exogenous activator BIM/BID induces cytochrome C release in DMSO-treated cells (Fig. 2m). As well, treatment with CR-1-31-B significantly enhanced cytochrome C release induced by BH3 peptides of BIM, BID and PUMA, in addition to the BCL2/BCL-XL inhibitor BAD, suggesting that apoptotic block in MOLM-14 is associated with enhanced expression of antiapoptotic BCL2 family members (e.g. Class C block) [58, 67], and that eIF4Ai can partially abrogate this effect (Fig. 2m). Therefore, the BH3 profiling results, together with the reduced expression of BCL2, BCL-XL and MCL1 that we observed, indicate that CR-1-31-B enhances apoptotic priming in MOLM-14 cells by decreasing the expression of antiapoptotic factors and therefore interferes with apoptotic blockage in these cells. Our data is consistent with previous reports showing that MOLM-14 cells show partial sensitivity to the BCL2 inhibitor venetoclax [68]. Enhanced apoptotic priming by CR-1-31-B was observed with inhibitors of BCL2 (e.g., BAD, venetoclax, ABT-737), in agreement with the synergy that we observed (Fig. 2k), versus only a marginal effect with inhibitors of MCL1 (NOXA, MS1) or BCL-XL (HRK) when these drugs were used as single agents. This shows MOLM-14 cells are largely dependent on BCL2, with smaller contributions from additional anti-apoptotic proteins. These results, together with western blots showing decrease in anti-apoptotic protein abundance, suggest that eIF4Ai impairs the anti-apoptotic adaptations of MOLM-14 cells and increases their vulnerability to BCL2 inhibition.

### EIF4Ai and araC have contrasting effects on mitochondrial fitness and bioenergetic profile

**Fig. 3:**
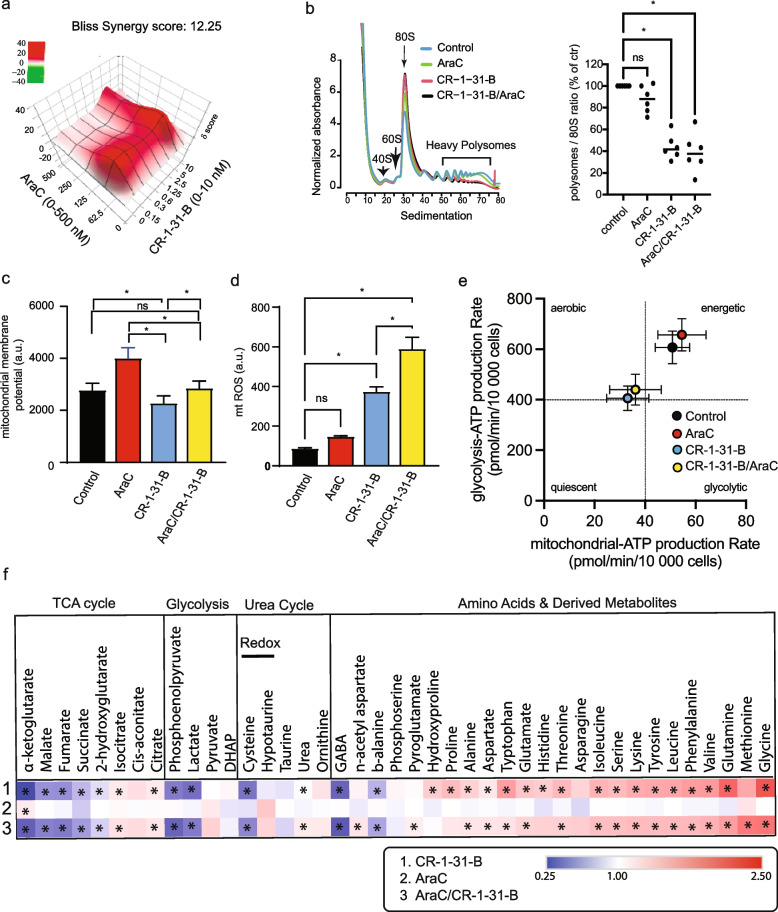
EIF4A inhibition and araC have contrasting effects on mitochondrial fitness and bioenergetic profile. AraC and CR-1-31-B synergize in MOLM-14 cells: **a** MOLM-14 cells were treated with CR-1-31-B (0-5 nM) in combination with araC (0-500 nM) for 24 h. Viability data was obtained using CellTiter-Glo and synergy between the drugs was assessed using the SynergyFinder application. CR-1-31-B hampers protein synthesis in the presence or the absence of araC: **b** (left) MOLM-14 cells treated with (i) vehicle, (ii) 250 nM araC (24 h), (iii) 10 nM CR-1-31-B (the last 1 h of treatment) and (iv) 250 nM araC (24 h) + 10 nM CR-1-31-B (the last 1 h of treatment). Sub-polysome, light-, and heavy-polysome fractions were obtained by ultracentrifugation using 5–50% sucrose gradients. During fractionation, UV absorbance at 254 nm (Abs 254 nm) was continuously monitored to obtain absorbance tracings. Position of 40S and 60S ribosomal subunits, monosome (80S) and polysomes are indicated. (right) The areas under the curve for the monosome (80S) and polysomes was used to calculate the polysome/80S ratio. AraC affects mitochondrial membrane potential in an eIF4A-dependent manner: **c** MOLM-14 cells were treated with vehicle, 2.5 nM CR-1-31-B, 250 nM araC, or their combination for 48 h. Mitochondrial membrane potential (Mitochondrial MP) and **d** mitochondrial superoxide (mtROS) content were quantified by flow cytometry using TMRE and mitoSOX, respectively; arbitrary units (a.u.). CR-1-31-B induces a shift towards a lower bioenergetic state: **e** MOLM-14 cells were treated with vehicle, 250 nM araC (48 h) and/or 2.5 nM CR-1-31-B (last 24 h of the 48 h incubation). The oxygen consumption rate (OCR) and extracellular acidification rate (ECAR) obtained using the Seahorse XF technology was used to calculate the ATP production rates by mitochondrial respiration (y-axis) and glycolysis (x-axis). Data related to Fig. 3e are shown in Supp. Fig. 2b-e. EIF4A inhibition and araC affect metabolite levels in MOLM-14 cells. **f** MOLM-14 cells were treated with vehicle, 250 nM araC (48 h) and/or 10 nM CR-1-31-B (treatment for the last 24 h of the total 48 h incubation with araC). Targeted analysis of metabolite content was done by GC/MS. The heatmap represents intracellular metabolites content relative to vehicle treated cells. Comparisons were assessed by two-way ANOVA followed by Šídák’s multiple comparisons test, *p < 0.05. Footnote. With the exception of targeted metabolite analysis (*n* = 2), the bars or circles represent the mean values of at least 3 independent experiments plus/minus the standard error of the mean (SEM) for c-d or the standard deviation for e. Comparisons between groups were assessed by One-way ANOVA followed by paired t-test, *p < 0.05 and ns indicates a *p* > 0.05. All functional analysis performed by flow cytometry were done for viable DAPI negative cells

We performed a similar combinatorial analysis of araC (0-500 nM) and CR-1-31-B (0-5 nM), which showed that the araC/CR-1-31-B drug combination reduces viability of MOLM-14 cells in a synergistic manner with a Bliss delta score of 11.9 (Fig. 3a). Because residual AML cells after araC treatment have been shown to upregulate mTORC1 signaling [18], we sought to investigate the effects of araC on mTORC1-dependent mRNA translation initiation. In vitro, treatment of MOLM-14 cells with an IC_25_ dose of araC did not affect the polysomes-to-monosome ratio (Fig. 3b). In contrast, CR-1-31-B reduced the heaviest polysome fractions by ~ 60% in presence or absence of araC-treated MOLM-14. These results show that CR-1-31-B reduces de novo protein synthesis in araC-treated MOLM-14 cells. Next, we assessed the effect of araC alone or in combination with CR-1-31-B on mitochondrial fitness. Interestingly, araC alone significantly enhanced MMP and this effect was counterbalanced by CR-1-31-B treatment (Fig. 3c). This suggests that, although eIF4A-dependent translation is not the primary driver of MMP in the absence of araC treatment, in our experimental setting, inhibition of eIF4A can abrogate araC-induced MPP changes. Furthermore, while not affecting mtROS at the concentration tested, araC enhanced CR-1-31-B-induced mtROS (Fig. 3d), suggesting a possible role for mtROS in the synergy between araC and CR-1-31-B [69, 70]. Noteworthy, the effects of araC on MMP occurred at a dose (IC_25_) that had a negligible effect on the rates of oxygen consumption, glycolysis or ATP synthesis in MOLM-14 cells (Supp. Fig. 2b-e). On the contrary, CR-1-31-B induced a significant reduction in ATP production from both oxidative phosphorylation and glycolysis, suggestive of an energetic crisis (Fig. 3e).

### EIF4Ai and araC reveal distinct metabolic vulnerabilities

To gain further insight into the impact of eIF4Ai and araC in metabolic reprogramming in AML, we searched for alterations in the intracellular content of metabolites of glycolysis and the TCA cycle, intracellular amino acids, and their key intermediates. We observed that CR-1-31-B treatment, with or without araC, resulted in reduced content of all TCA cycle intermediates, except for citrate, cis-aconitate and isocitrate (Fig. 3f). Low levels of TCA intermediates are in agreement with reduced oxygen consumption and ATP production rates in CR-1-31-B-treated MOLM-14 cells (Fig. 2e-f). Reduced phosphoenolpyruvate, pyruvate and lactate levels by CR-1-31-B are in line with the reduced glycolysis in MOLM-14 cells observed with Seahorse (Fig. 2d). In addition, CR-1-31-B led to the accumulation of most amino acids, an effect that could be associated with halted protein synthesis but has also been associated with inhibition of mitochondrial respiration [71]. In contrast, araC treatment resulted in the accumulation of α-ketoglutarate (α-KG) with a modest reduction of amino acid content, suggesting increased α-KG production from amino acids (TCA cycle anaplerosis). In line with its negligible effect on the rates of oxygen consumption, glycolysis or ATP synthesis (Supp. Fig. 2b-e), araC at the concentration tested (IC_25_) did not significantly impact the majority of measured metabolites in MOLM-14 cells.

### EIF4Ai reduces leukemic burden in vivo and is well tolerated

**Fig. 4:**
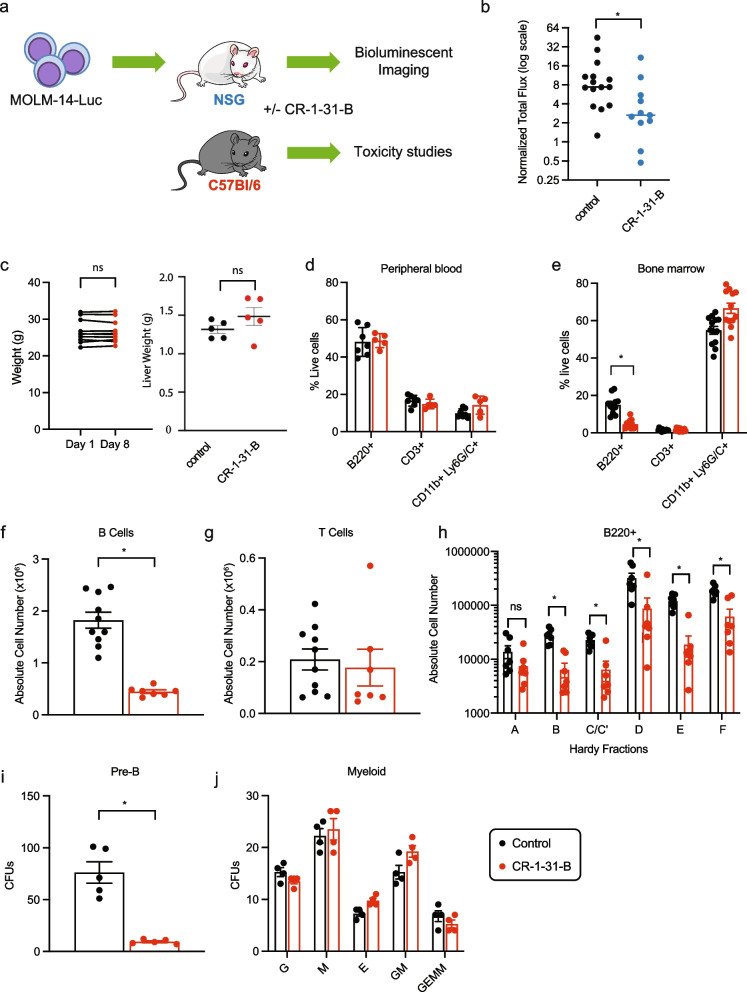
EIF4Ai is well tolerated and reduces leukemic burden in vivo. **a** The effect of CR-1-31-B was tested at day 10 post-transplantation of MOLM-14-Luc cells. CR-1-31-B toxicity was assessed at day 7 in healthy C57BL/6 J mice treated with 0.2 mg/kg CR-1-31-B (red) or vehicle (control, black), except for body weight. **b** Bioluminescent intensities of NSG mice transplanted with MOLM-14-Luc on Day 8 normalized to day 1 intensity. The bars represent the median values, * *p* < 0.05, unpaired t-test. **c** Body weight (left panel) and liver weight (right panel) of healthy C57BL/6 J mice treated with CR-1-31-B on days 1 and 8 or 8 days of treatment respectively. **d**, **e** Fractions of B220^+^ B-cells, CD3^+^ T-cells and CD11b^+^Ly6G/C^+^ myeloid cells in **d** the peripheral blood and **e** the bone marrow of vehicle and CR-1-31-B treated mice. **f**-**h** Absolute number of **f** B220^+^ B-cells, **g** CD3^+^ T-cells and **h** cells contained within each Hardy Fraction in CR-1-31-B and vehicle treated mice within the BM. **i**-**j** Colony forming units (CFUs) for **i** pre-B cells and **j** myeloid progenitors from vehicle and CR-1-31-B treated bone. Data related to Fig. 4 are shown in Supp. Fig. 3*.* Footnote: In all graphs except for **d** mean values with SEM are represented, *p < 0.05 and ns indicates a *p* > 0.05, paired t-test

The effect of CR-1-31-B on MOLM-14 leukemic growth in vivo was tested using bioluminescence as a readout, with a dose of CR-1-31-B (0.2 mg/kg/day) reported to be active in a xenograft model of pancreatic cancer [31]. In addition, we performed toxicity studies in immunocompetent C57Bl/6 mice (Fig. 4a). We injected luciferase-expressing MOLM-14 cells in NSG recipients and, 10 days later, started treatment with daily intraperitoneal injections of CR-1-31-B for 7 days. Treatment with CR-1-31-B significantly reduced the growth of the MOLM-14 cells (Fig. 4b). Importantly, the same dose was well tolerated in C57BL/6 mice with no significant weight loss, change in activity, or liver weight compared to vehicle-treated controls (Fig. 4c). To further evaluate the possible toxicity of CR-1-31-B on the normal hematopoietic system, we analysed the impact on the hematopoietic lineages in the blood, bone marrow, and spleen (Supp. Fig. 3a). CR-1-31-B has no major effect on cell death or cellularity of the bone marrow (Supp. Fig. 3b), or peripheral blood lineages (Fig. 4d), but affects bone marrow B-cell development (Fig. 4e). Specifically, we observed a significant reduction in bone marrow B220^+^ cells (Fig. 4f) but not T cells (Fig. 4g). To further delineate the effects of CR-1-31-B on different stages of B-cell development, we examined the distinct populations of B-cell precursors within the BM (known as Hardy fractions) [72] as well as the ability of bone marrow hematopoietic progenitors to differentiate in vitro using colony-forming unit assays. Hardy Fractions represent increasing stages of maturation and range from fraction A (Pre-Pro B cells) to fraction F (Mature B-cells) (Supp. Fig. 4). We found a significant block in Hardy fractions B-F (Fig. 4h) and a trend towards decrease of fractions A, accompanied by a reduction in the pre-B cell colonies in methylcellulose (Fig. 4i). This and the observation of decreased common lymphoid progenitor cells (CLPs) (Supp. Fig. 3c, d) suggests that eIF4A is important for B-lymphopoiesis at an early stage of lymphoid lineage commitment. Importantly, this daily treatment for 7 days did not decrease the fractions of circulating mature B cells (Fig. 4d), the number of myeloid colony-forming units within the bone marrow (Fig. 4j), or the number of phenotypically defined hematopoietic progenitors and stem cells (HSPCs; Supp. Fig. 3c, d). Overall, these in vivo findings support the existence of a therapeutic window for eIF4A targeting of AML cells while preserving normal myelopoiesis.

## Discussion

Our work provides new insights into the relationship between AML chemotherapy resistance and mTORC1 signaling, by demonstrating that eIF4F-dependent mRNA translation contributes to preservation of mitochondrial functionality and is essential for the maintenance of redox homeostasis, mitochondrial OX/PHOS, glycolysis, and balance between apoptotic regulators. This indicates that eIF4Ai such as CR-1-31-B hold the potential to synergize with therapeutic agents enhancing redox stress, compromising bioenergetics, or inducing apoptosis.

AML pathogenesis and resistance to therapy is associated with metabolic rewiring [41, 73] of amino acids, redox control, glucose, and fatty acid metabolism [19, 38, 73–79]. mTORC1 dysregulation has been associated with de novo and acquired resistance to both chemotherapy and targeted therapies [18–26] and recent studies suggest that residual human AML cells after treatment with araC are characterized by elevated mTORC1 signaling and altered mitochondrial functions [18, 39, 80]. From a transcriptional standpoint, we previously showed that subsets of AML cases with enhanced expression of mTORC1 signature genes have worse clinical outcomes [18]. Here, we extended our previous results using the mouse syngeneic KMT2A/MLLT3 model (FM4), in which we showed that mTORC1 is upregulated in the context of chemoresistance in vivo [18], by demonstrating concomitantly increased rates of protein synthesis and mitochondrial function.

To investigate whether eIF4Ai may be beneficial to rescue the poor response to therapy driven by mTORC1 activity, we performed studies in the chemoresistant human MOLM-14 model, harboring a KMT2A/MLLT3 fusion but also a FLT3-ITD mutation that constitutively activates mTORC1 signaling, which was established from the peripheral blood of a patient at relapse of AML [12, 49]. In contrast to the FM4 model, chemotherapy failed to affect mTORC1 activity in MOLM-14 cells in vivo or in vitro. While others have shown that araC can enhance mTORC1 activity in AML lines in vitro through hyperactivation of mutant FLT3 in an autocrine or paracrine manner [81, 82], it is possible that in our setting, the lack of mTORC1 activation might relate to the use of the lower concentrations of araC. Likewise, in vivo araC treatment failed to affect mTORC1 targets and MMP in MOLM-14 cells. While genetic differences preclude direct comparisons between the FM4 and MOLM-14 models, we speculate that in vivo chemotherapy in the MOLM-14 model did not affect mTORC1 signaling and MMP because it did not have an antineoplastic effect at the maximal tolerable dose used [39], which may be required to trigger adaptive responses in AML cells. Nevertheless, the sensitivity of MOLM-14 cells to eIF4Ai as single-agent in vitro and in vivo highlights that the mTORC1/eIF4F pathway is constitutively active and essential. Interestingly, although araC (IC_25_) did not enhance de novo protein synthesis in MOLM-14, inhibition of eIF4A synergized with araC-induced cytotoxicity, suggesting that cap-dependent translation is involved in promoting AML survival under chemotherapy stress.

Our results are in agreement with previous studies in AML showing that mTORC1 drives glucose metabolism and its inhibition abrogates glycolytic flux [19, 83, 84]. Specifically, inhibition of mTORC1 or silencing of Raptor expression reduces pyruvate dehydrogenase kinase 1 expression and the use of pyruvate derived carbon through the TCA cycle [19]. Further, it was found that suppression of mTORC1 enhances oxidative stress because of reduced glucose flux through the pentose phosphate pathway, which is essential for glutathione regeneration [19]. Our finding of markedly decreased glycolysis after eIF4Ai treatment suggests that the metabolic program driven by mTORC1 in AML cells comprises a translational eIF4A-dependent component. In line with our results in MOLM-14 cells, in pancreatic cancer, eIF4Ai impairs both oxidative phosphorylation and glycolysis through altered synthesis of mitochondrial respiration chain components and glucose transporters, therefore forcing the use of glutaminolysis, whereby glutamine-derived α-KG is used to feed the TCA cycle [31].

Our results reveal that eIF4A inhibition has a profound effect on the bioenergetic capacity of MOLM-14 AML cells and indicates that eIF4A sustains anabolic metabolism, as outlined above and in the study by Chan et al. [31]. This study further proposed that CR-1-31-B impairs ROS scavenging through enhanced use of glutamine for TCA anaplerosis at the expense of glutathione synthesis [31]. The partial rescue of CR-1-31-B-induced apoptosis using ROS scavengers highlights the role of redox stress in mediating cell death in MOLM-14 after eIF4A inhibition. In AML cells, impaired redox control by cysteine depletion results in dysfunction of the ETC complex II, highlighting the vulnerability of AML cells to redox stress [85]. Therefore, eIF4Ai might be cytotoxic in AML through an interplay between redox homeostasis and bioenergetics.

Importantly, co-treatment with araC and CR-1-31-B demonstrates synergy in killing of AML cells. Our results show that araC enhances MMP, and CR-1-31-B abrogated this effect, demonstrating that chemotherapy activates eIF4A-dependent proteins impacting mitochondrial functions such as membrane potential and bioenergetics. Rewiring of central carbon metabolism by araC has been associated to altered amino acid metabolism [80], glucose consumption and fatty acid metabolism [39]. Enhanced α-KG content might result from rewiring of amino acid metabolism, such as glutamine/glutamate, to feed the TCA cycle. In agreement with this possibility, the content of glutamine, glutamate, proline, and histidine, all glucogenic amino acids, is slightly reduced in araC-treated MOLM-14 cells. Importantly, exacerbated mtROS by araC co-treatment might further prime MOLM-14 cells to CR-1-13-B-induced apoptosis.

It is possible that eIF4Ai further contributes to AML cell death by reducing levels of the anti-apoptotic proteins BCL2, BCL-XL and MCL1 that control mitochondrial function and integrity [86]. In AML, BCL2 also contributes to maintenance of mitochondrial bioenergetics and redox homeostasis [38, 86] by favoring the closure of the mPTP [80, 85–87]. In MOLM-14 cells, we found a strong synergy between the BCL2 inhibitor venetoclax and CR-1-31-B in vitro, suggesting that eIF4A limits apoptosis. Using dynamic BH3 profiling, a technique that leverages pro-apoptotic peptides or inhibitors of anti-apoptotic proteins with different specificities to assess apoptotic blocks, our results suggest that eIF4Ai shifts the balance of MOLM-14 cells towards apoptosis by abrogating the excess of anti-apoptotic proteins (“type C block”) [58, 67]. Whether or not eIF4A-mediated regulation of BCL2, BCL-XL and MCL1 expression occurs at the translational level in our settings remains to be determined, but this has been previously shown in other cellular models [34, 53, 88]. In addition to decreased expression of anti-apoptotic proteins, it is possible that eIF4Ai potentiates apoptosis through the induction of cellular stresses such as ROS or impaired bioenergetics. Regarding response to BCL2-family inhibitors, AML samples are highly heterogeneous. Therefore, the individual contributions of these specific triggers and downstream effectors to the overall pro-apoptotic effect of eIF4Ai remain to be defined in a range AML genotypes. However, in MOLM-14 cells, we expect that enforcing expression of BCL2 or other family members would rescue the phenotype to some extent.

In vivo, CR-1-31-B was effective in reducing leukemic burden in the aggressive and araC-resistant MOLM-14 AML xenotransplantation model. Importantly, MOLM-14 cells express constitutively active mutant FLT3 and therefore display elevated mTORC1 signaling [89], possibly rendering them functionally addicted to eIF4A-driven processes. The in vivo anti-leukemic activity of CR-1-31-B as a single agent strongly supports the further therapeutic assessment of eIF4Ai. Notably, the fact that an anti-leukemic regimen of CR-1-31-B was well tolerated by healthy mice, without peripheral blood cytopenias or impairment of normal myeloid progenitors, suggests the existence of a therapeutic window. Although CR-1-31-B decreases common lymphocyte and B-progenitor numbers, from a therapeutic standpoint, agents that selectively target B-cells (such as the monoclonal antibody rituximab) are overall well tolerated and B-cell function can be compensated by transfusion of intravenous immunoglobulins. Furthermore, our results anticipate that eIF4Ai, including CR-1-13-B, hold the potential to curtail mTORC1 driven persistence to therapeutic agents in the context of specific oncogenic mutations that drive constitutive signaling (*FLT3*, *RAS*, *KIT*). In the context of FLT3 inhibitors, compensatory activation of mTORC1 signaling, through diverse upstream pathways, is essential for survival and relapse [79]. Noteworthy, the favorable toxicity of eIF4A inhibition has led to Phase I-II clinical trials assessing the effect of zotatifin, for advanced solid tumors (NCT04092673) and mild Covid 19 cases (NCT04632381).

## Conclusion

Our findings using the pharmacological eIF4Ai CR-1-13-1-B in the chemoresistant MOLM-14 model of AML are consistent with the pleotropic effects of the mTORC1/eIF4F/eIF4A axis in promoting bioenergetic and anti-apoptotic programs in cancer cells and uncover a key role of eIF4A in facilitating metabolic rewiring and maintaining mitochondrial homeostasis and bioenergetics. The impact of the in vivo bone marrow microenvironment, including hypoxic gradients, on these key processes remains to be investigated. Nevertheless, CR-1-31-B administered as a single agent was effective in significantly reducing growth in the MOLM-14 model in vivo. As a proof of concept, we used a model with constitutively activated mTORC1 to target downstream effectors. Therefore, additional studies in human patient samples are required to reveal whether eIF4Ai can be applied as a strategy to target mTORC1-dependent AML growth across the diverse genotypes that characterize human diseases and/or define biomarkers predictive of response. Our expectation is that our present work will promote further studies validating the role of eIF4A in leukemogenesis and the therapeutic potential of pharmacological eIF4A inhibition.

## Supplementary Information

**Additional file 1: Supplementary Fig. 1.** a. Gating strategy to assess the effect of chemotherapy on live AML cells defined using viability 700 stain/FSC-A and CD45.1/FSC-A in mononuclear cells isolated from the bone marrow of mice treated with vehicle (Control, upper row) or chemotherapy (Treated, middle row). The lower row shows unstained controls b. The effect of chemotherapy on p4E-BP, pS6, TMRE or Mitosox staining was assessed in live AML cells. **Supplementary Fig. 2.** a. Quantification of band intensities for western blots in Fig. 2j was done using ImageJ software (https://imagej.nih.gov/ij/index.html). Quantification was performed from 3 independent experiments and data for BCL2, BCL-XL and MCL1 was normalized by actin levels for each experiment. Data is expressed as average +/− SD, relative to DMSO control. * *p* < 0.05. b-f. MOLM-14 cells were treated with DMSO, 250 nM araC for 48 h, and/or 2.5 nM CR-1-31-B for 24 h. Oxygen consumption (OCR) and extracellular acidification rate (ECAR) measurements are shown in b and d, respectively, and were conducted in an XFe96 Seahorse extracellular flux analyzer and normalized to cell counts in the presence of different stressors. Oligomycin is an inhibitor of mitochondrial complex V (ATP synthase), FCCP is an uncoupler that dissipates mitochondrial proton gradient, Rotenone and Antimycin a (Aa) are inhibitors of Complex I and II, respectively; 2DG is a glucose analogue that inhibits the first step of glycolysis. OCR and ECAR rates were utilized to calculate c oxidative phosphorylation, e glycolytic parameters, and f to estimate the contribution of OxPhos and glycolysis to ATP production. The results represent 3 independent experiments and SEM; ns indicates *p* > 0.05 and * *p* < 0.05. **Supplementary Fig. 3.** a. BM cells from vehicle (control, black bars) or CR-1-31-B treated mice (0.20 mg/kg i.p. daily for 7 days, red bars) were stained with acridine orange/propidium iodide. The bars represent mean absolute cell numbers and SEM, ns indicates *p* > 0.05. b. Gating strategy for the evaluation of hematopoietic lineages for B cells (B220+), helper T cells (CD4^+^), cytotoxic T cells (CD8^+^) and myeloid cells (CD11b + GR-1+). c. Gating strategy for hematopoietic stem and progenitor cells (HSPC) in viable, lineage-negative (Lin^−^) cells: Sca-1^+^ and c-kit^+^ cells (LSK cells), granulocyte-macrophage progenitors (GMPs, CD16/32^+^CD34^+^Lin^−^Sca-1^−^c-kit^+^), common lymphoid progenitor (CLPs, CD127^+^/LSK cells). Lymphoid myeloid progenitors (LMPPs) and HSPC were discriminated by differential expression of CD135 in LSK cells, HSCs enriched within the HSPC gate as CD48^−^CD150^+^ (LSK SLAM). d Effect of vehicle (control, black bars) or CR-1-31-B 0.20 mg/kg i.p. daily for 7 days (red bars) in defined HSPC populations. The results are expressed as absolute cell number means and SEM. Differences were assessed by t-test, *p < 0.05. **Supplementary Fig. 4.** Lymphocyte suspensions from bone marrow were stained with the following mouse specific antibodies: PerCP-Cy5.5-anti-B220 (RA3-6B2; BD), PE-Cy7-anti-CD43 (S7; BD), PE-anti-Ly-51(BP-1; BD), FITC-anti-CD24(M1/69; BD), BV421-anti-IgM (R6-60.2; BD) and BV605-anti-IgD (11-26c.2a; BD). Samples were acquired on LSRFortessa (BD) and data were analyzed with FlowJo software (Treestar). **Supplementary Fig. 5.** a. Representative gating strategy for BH3 profiling analysis. Cells were gated first on size, followed by elimination of cell doublets (FSC-H vs FSC-A). Dead cells were removed via staining with Aqua Live/Dead (Invitrogen, #L34966). Lastly, cytochrome C retention was measured using AF647 (BD Biosciences, #558709). Alamethicin acts as the negative staining control, as no cells retain cytochrome C, while DMSO acts as the positive staining control. Gates for cytochrome C were set to the negative control (Ala) for each experiment. b. Interaction partners of BH3 peptides and pharmacological inhibitors. The table shows the binding schematic between peptides/inhibitors used in the dynamic BH3 profiling experiments and the BCL2 family of proteins (BCL2, MCL1, BCL-XL). BIM/BID are targeted by all BCL2 family proteins, while PUMA binds them in order to free BIM/BID for apoptotic activation. BAD, ABT-737, and venetoclax primarily target BCL2. Peptides MS1 and NOXA, as well as inhibitor S63845, target MCL1. HRK and inhibitor A-1331852 selectively target BCL-XL.

## Data Availability

The data and cell lines generated during the current study are available from the corresponding authors on reasonable request.

## Abbreviations

4E-BP1: Eukaryotic translation initiation factor 4E-binding protein 1
a.u.: Arbitrary units
AML: Acute myeloid leukemia
α-KG: Alpha-ketoglutarate
AraC: Cytosine arabinoside
BCL2: B-cell lymphoma 2
BCL-XL: B-cell lymphoma-extra large
BM: Bone marrow
CFUs: Colony forming units
DHAP: Dihydroxyacetone phosphate, formerly glycerone phosphate
ECAR: Extracellular acidification rate
eIF4A, eIF4E, eIF4G eIF4F: Eukaryotic initiation factor-4A, E, G, and F, respectively
EIF4Ai: Eukaryotic initiation factor-4A inhibitor
ETC: Electron transport chain
FLT3: Fms-like tyrosine kinase 3
GC/MS: Gas chromatography–mass spectrometry
i.p.: Intraperitoneally
IC_25_ and IC_50_: 25 and 50% inhibitory concentrations, respectively
IDH1/2: Isocitrate dehydrogenase
MCL1: Myeloid cell leukemia-1
MFI: Mean fluorescence intensity
mTORC: Mammalian target of rapamycin complex
MMP: Mitochondrial membrane potential
mPTP: Mitochondrial permeability transition pore
OCR: Oxygen consumption rate and
OX/PHOS: Oxidative phosphorylation
PCD4: Programmed cell death protein 4
PDX: Patient-derived xenograft
ROI: Regions of interest
ROS & mtROS: Reactive oxygen species and mitochondrial reactive oxygen species, respectively
rpS6 (S6): Ribosomal protein S6
S6K: Ribosomal protein S6 kinase
TCA: Tricarboxylic acid cycle
