# Supplementary material for: EIF4A inhibition targets bioenergetic homeostasis in AML MOLM-14 cells in vitro and in vivo and synergizes with cytarabine and venetoclax
Source: J Exp Clin Cancer Res. 2022 Dec 9;41:340. doi: 10.1186/s13046-022-02542-8 (PMC9733142; doi:10.1186/s13046-022-02542-8)
Supplement: Supplementary file 1 — Additional file 1: Supplementary Fig. 1. a. Gating strategy to assess the effect of chemotherapy on live AML cells defined using viability 700 stain/FSC-A and CD45.1/FSC-A in mononuclear cells isolated from the bone marrow of mice treated with vehicle (Control, upper row) or chemotherapy (Treated, middle row). The lower row shows unstained controls b. The effect of chemotherapy on p4E-BP, pS6, TMRE or Mitosox staining was assessed in live AML cells. Supplementary Fig. 2. a. Quantification of band intensities for western blots in Fig. 2j was done using ImageJ software (https://imagej.nih.gov/ij/index.html). Quantification was performed from 3 independent experiments and data for BCL2, BCL-XL and MCL1 was normalized by actin levels for each experiment. Data is expressed as average +/− SD, relative to DMSO control. * p < 0.05. b-f. MOLM-14 cells were treated with DMSO, 250 nM araC for 48 h, and/or 2.5 nM CR-1-31-B for 24 h. Oxygen consumption (OCR) and extracellular acidification rate (ECAR) measurements are shown in b and d, respectively, and were conducted in an XFe96 Seahorse extracellular flux analyzer and normalized to cell counts in the presence of different stressors. Oligomycin is an inhibitor of mitochondrial complex V (ATP synthase), FCCP is an uncoupler that dissipates mitochondrial proton gradient, Rotenone and Antimycin a (Aa) are inhibitors of Complex I and II, respectively; 2DG is a glucose analogue that inhibits the first step of glycolysis. OCR and ECAR rates were utilized to calculate c oxidative phosphorylation, e glycolytic parameters, and f to estimate the contribution of OxPhos and glycolysis to ATP production. The results represent 3 independent experiments and SEM; ns indicates p > 0.05 and * p < 0.05. Supplementary Fig. 3. a. BM cells from vehicle (control, black bars) or CR-1-31-B treated mice (0.20 mg/kg i.p. daily for 7 days, red bars) were stained with acridine orange/propidium iodide. The bars represent mean absolute cell numbers and SEM, [file 13046_2022_2542_MOESM1_ESM.pdf]

Supplementary Figure 1

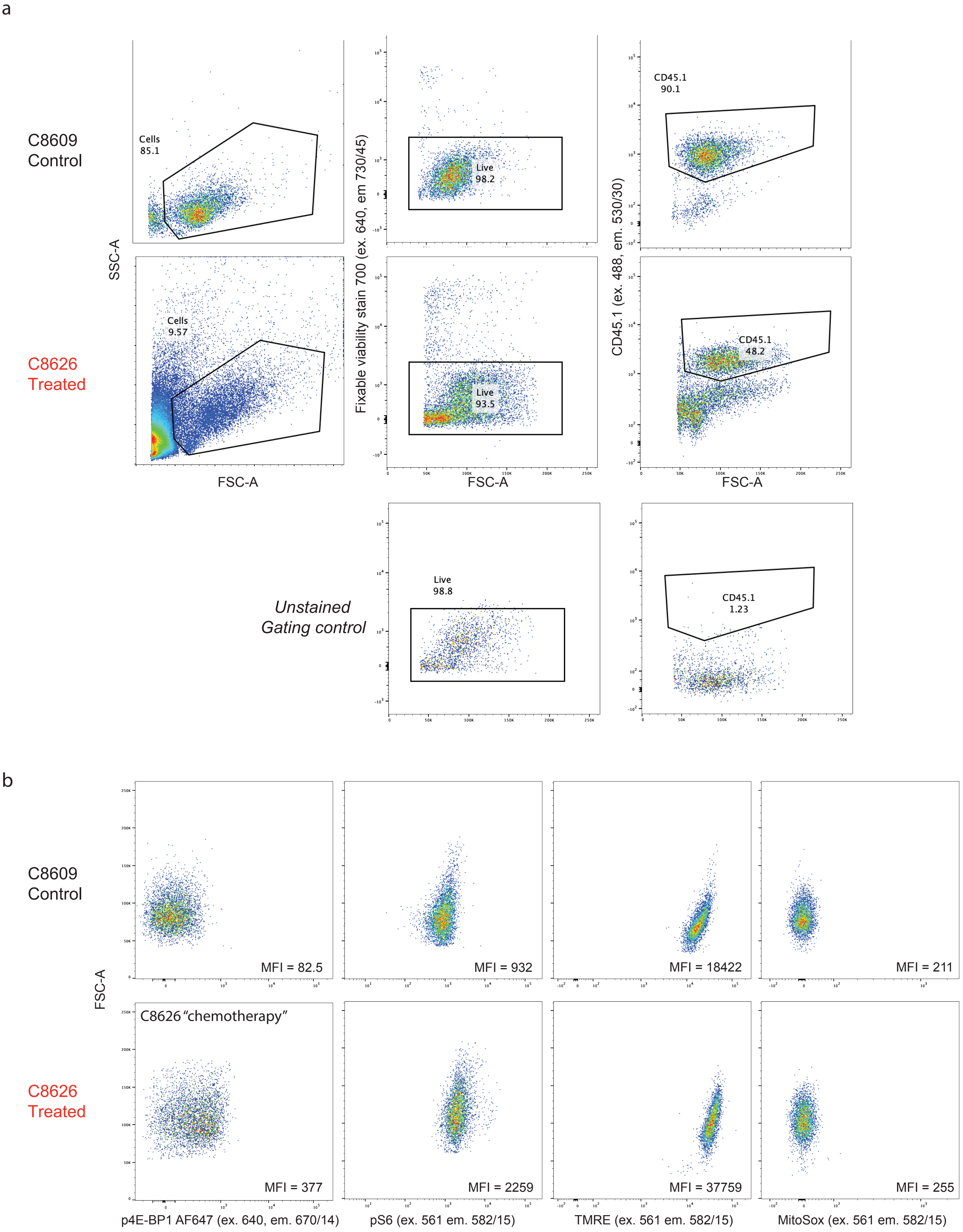

Supplementary Figure 2

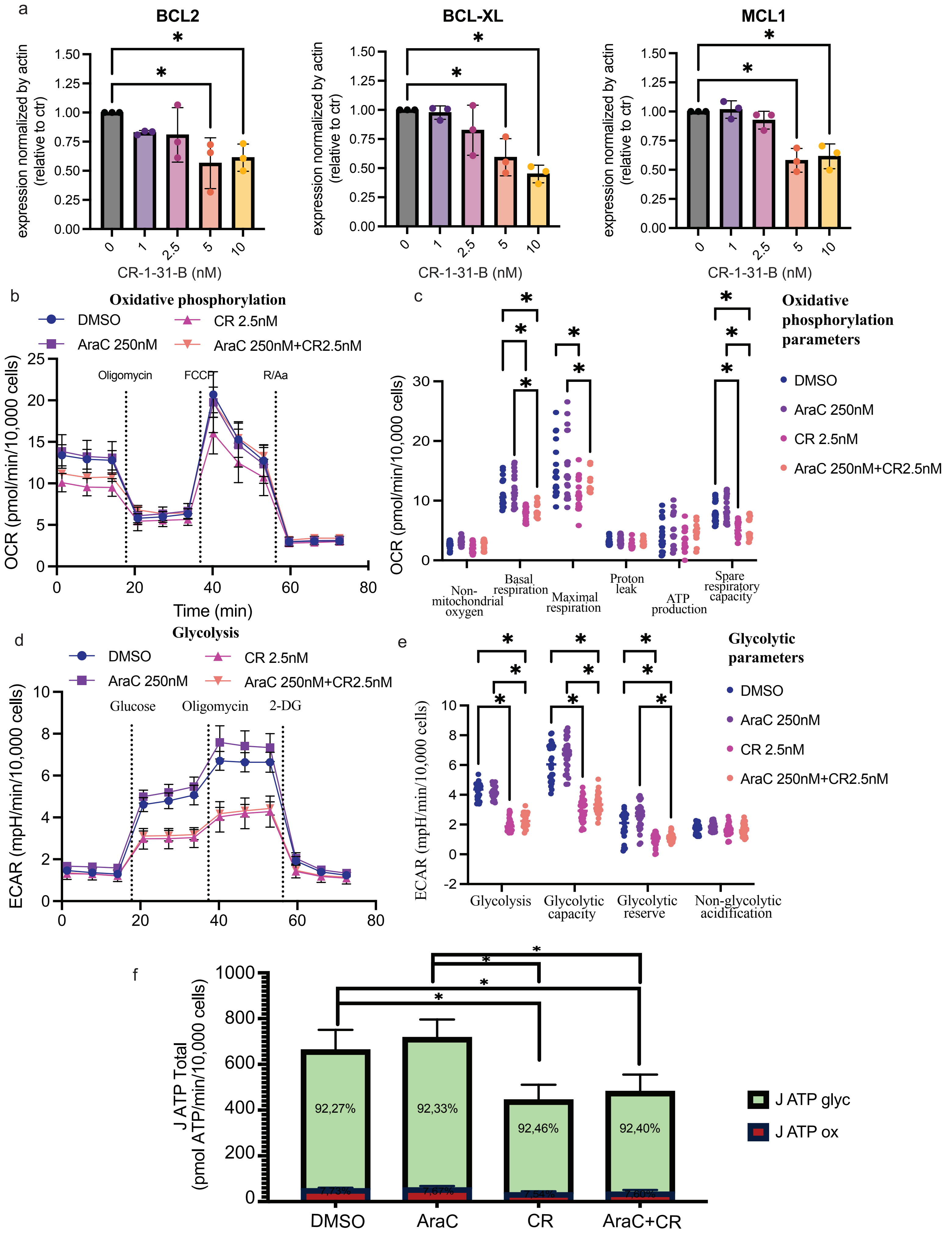

Supplementary Figure 3

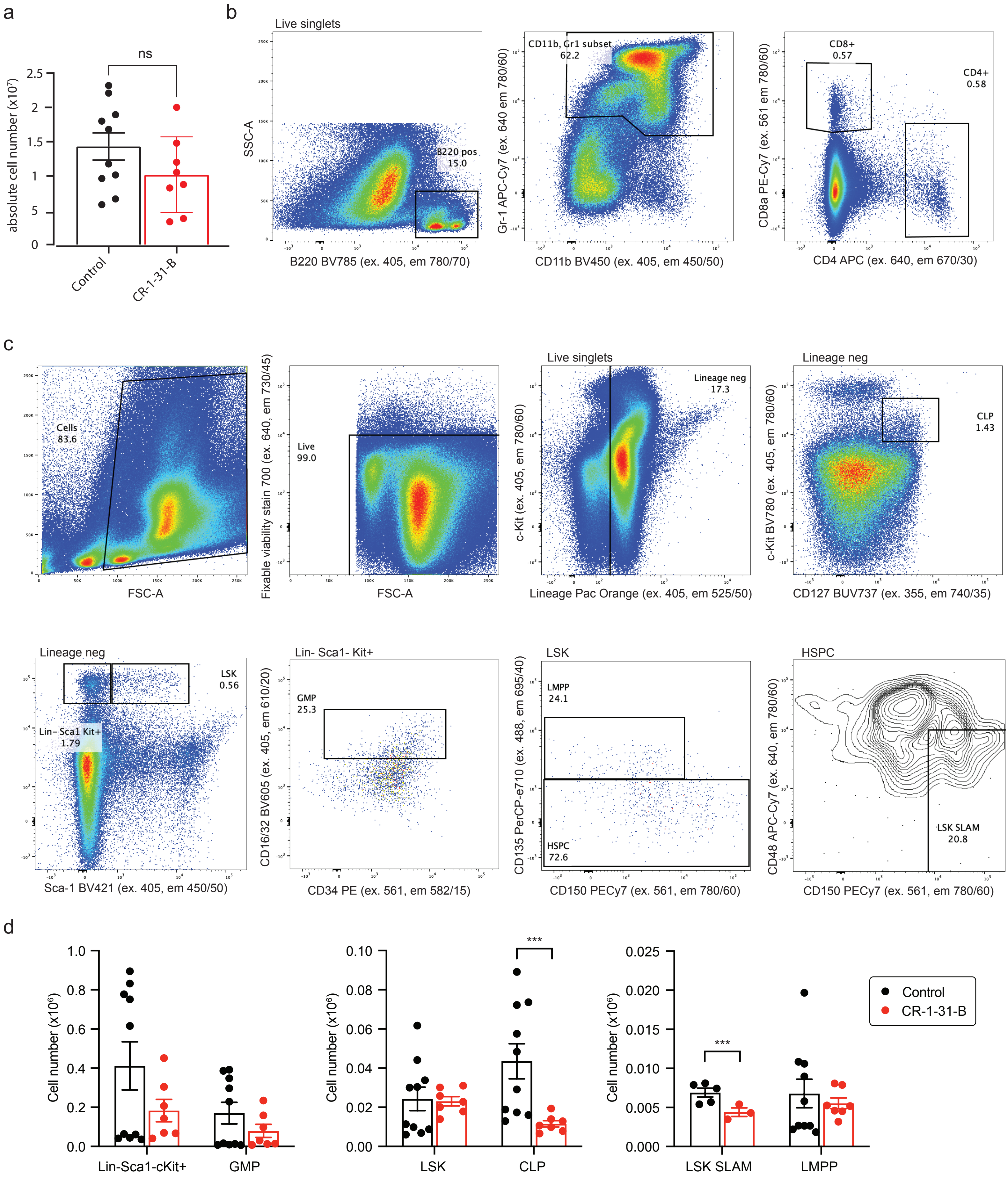

Supplementary Figure 4

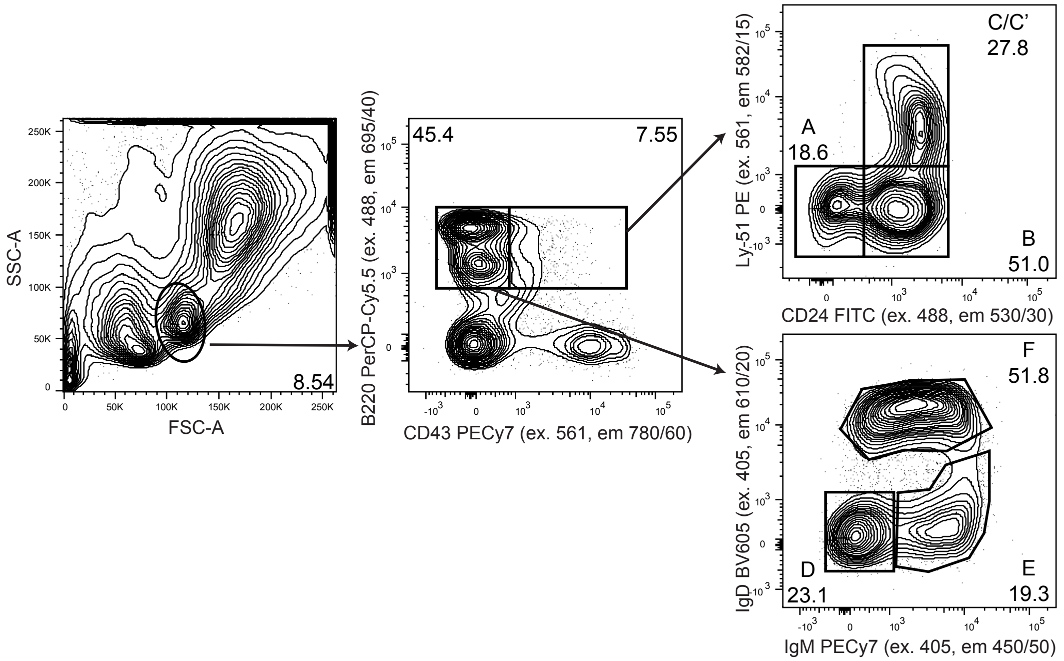

Supplementary Figure 5

a

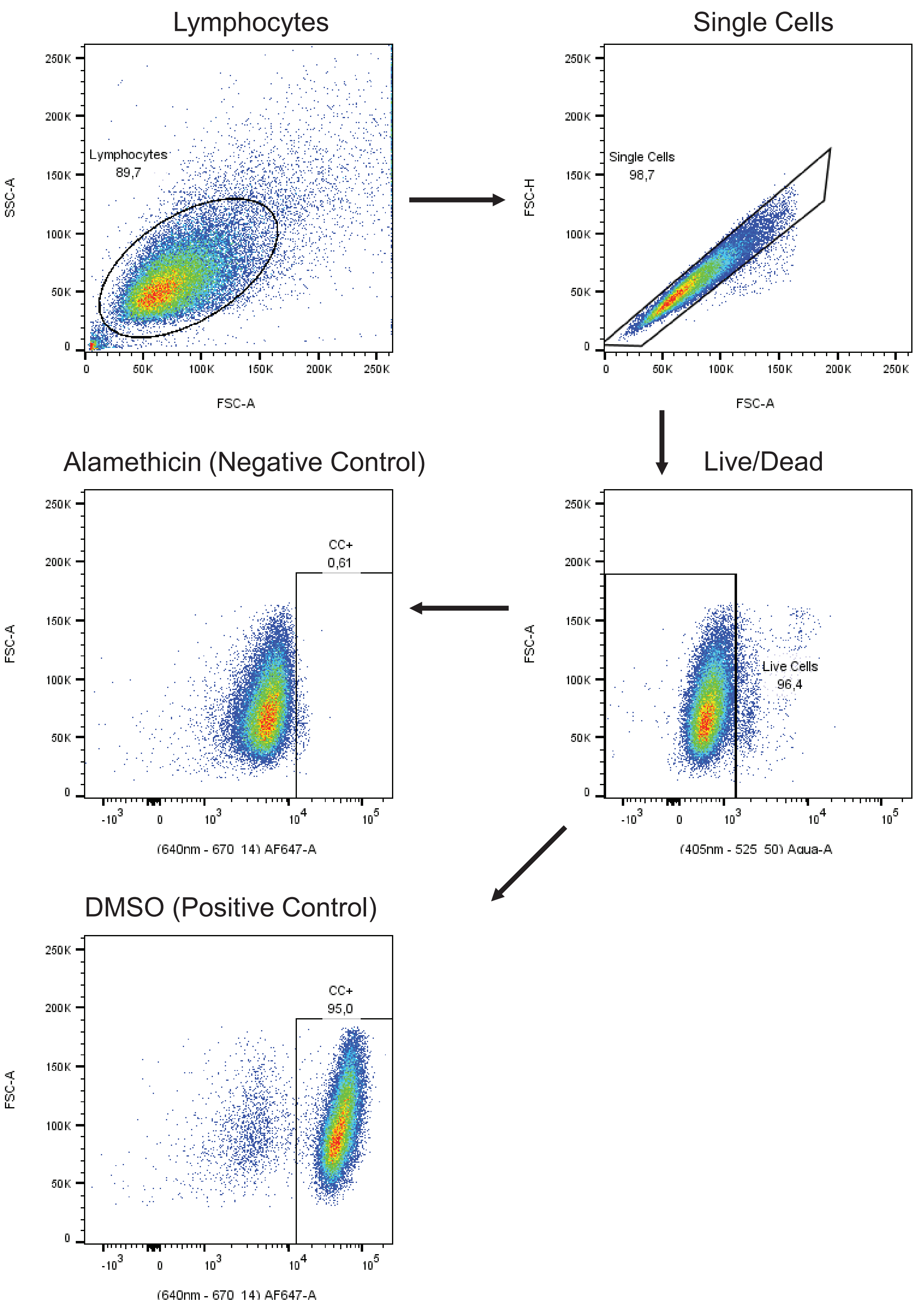

b

	BIM	BID	PUMA	BAD	NOXA	MS1	HRK	ABT-737	Venetoclax	A-1331852	S63845
BCL2											
MCL1											
BCLXL											
